# Effect of osseodensification on the increase in ridge thickness and the prevention of buccal peri-implant defects: an in vitro randomized split mouth pilot study

**DOI:** 10.1186/s12903-022-02242-x

**Published:** 2022-06-13

**Authors:** Fausto Frizzera, Rubens Spin-Neto, Victor Padilha, Nicolas Nicchio, Bruna Ghiraldini, Fábio Bezerra, Elcio Marcantonio

**Affiliations:** 1 FAESA University Center, Vitória, Brazil; 2 Aarhus University, Department of Dentistry and Oral Health, Aarhus, Denmark; 3 Brazilian Dental Association at Espírito Santo, Serra, Brazil; 4 São Paulo State University (UNESP), School of Dentistry, Department of Diagnostic and Surgery, Araraquara, Brazil; 5 Dental Research Division, School of Dentistry, Paulista University, São Paulo, Brazil; 6 São Paulo State University (UNESP), Department of Chemical and Biological Sciences, Institute of Biosciences, Botucatu, Brazil

**Keywords:** Osseodensification, Bone augmentation, Prevention, Dental implants, Alveolar ridge expansion

## Abstract

**Background:**

Implant installation with conventional drilling can create buccal bone defects in areas of limited ridge thickness. Implant installation with osseodensification may aid in preventing buccal bone defects in these situations. This in vitro pilot study evaluated the impact of osseodensification on the increase in alveolar ridge thickness and the prevention of buccal peri-implant defects.

**Methods:**

Ten fresh pig mandibles with limited bone thickness were selected for use in an experimental randomized split mouth pilot study. Two site-preparation protocols were used: conventional drilling with cutting burs (CTL, n = 10) and osseodensification with Densah® burs (OD, n = 10). After implant bed preparation, 20 implants (4.5 × 10 mm) were placed in the prepared sites and the insertion torque was recorded. Clinical and photographic analysis evaluated ridge thickness and the extent (height, width, and area) of bone defects in the buccal and lingual bone walls following implant placement. Three-dimensional measurements were performed using STL files to analyze the increase in buccal ridge thickness following site preparation and implant placement. The height of the buccal bone defect was considered as the primary outcome of this study. Defect width, area, implant insertion torque, and linear buccal ridge increase after implant site preparation and installation were also assessed. Non-parametric evaluations were carried out with the Mann–Whitney test to verify intergroup differences.

**Results:**

There was no statistically significant difference between groups in the baseline ridge thickness. OD presented a significantly higher insertion torque, associated with reduced buccal and lingual bone defect width, in comparison to CTL.

**Conclusions:**

The increase in buccal ridge thickness after site preparation and implant placement was significantly higher in OD compared to CTL. Osseodensification increased the ridge thickness through expansion and reduced buccal bone defects after implant installation.

## Background

Dental implants are a reliable and well-documented solution for replacing missing teeth. Patients prefer implant-supported restorations that are similar to their natural dentition. This preference has increased the complexity of implant treatment, making it necessary to reestablish the harmony between the prosthetic crowns and the surrounding hard and soft tissues. To achieve an adequate esthetic and functional outcome, the implant must be installed in the correct tridimensional position, an action that often requires additional bone preservation and/or reconstruction procedures [1, 2].

The alveolar process is remodeled after tooth extraction and promotes changes in the shape of the ridge, which can complicate or preclude implant installation without previous bone grafting [3]. Bone grafts have been extensively used to provide sufficient anchorage prior to implant placement [4] or to correct small peri-implant defects at the time of implant installation [5, 6]. Although documented as having good predictability, bone grafting increases the cost and time of treatment, surgical morbidity, and consumption of postoperative medications while decreasing patient acceptance [7].

Evaluating the results of a therapy considering patient-centered outcomes, along with the clinical and biological rationale, enable less invasive procedures with reduced treatment time [8]. Surgical and prosthetic techniques that promote an increase in hard and soft tissue quantity and quality are interesting options to guarantee more favorable results for patients [9].

The osseodensification protocol, initially proposed by Huwais [10, 13], represents a paradigm shift in the preparation of bone tissue prior to implant placement. It has shown promising results in the osseointegration process [11], enabling to increase the bone density at the prepared implant site [12, 13], avoiding more invasive techniques to elevate the maxillary sinus membrane [14], and increasing the volume of the ridge [15], preventing the occurrence of peri-implant bone defects.

The current protocol for the preparation of the implant site consists of using cutting drills at high speed and with clockwise rotation under constant irrigation to remove the bone tissue and install the implant in the desired region [16]. Osseodensification uses non-cutting drills in counter clockwise rotation to prepare the site to receive an implant. The prepared bone fragments are reintroduced to the site's lateral trabecular bone walls, compacting and increasing the density of the bone instead of removing the bone as performed in conventional drilling. Biologically, osseodensification provides greater bone-to-implant contact and increases the removal torque of implants, besides reducing the time required for osseointegration [11, 12, 15, 17]. From the clinical point of view, osseodensification promotes greater primary stability [18]. The implants installed using this technique can also have a larger diameter, when compared to the conventional technique of osteotomy using cutting drills, and can increase the volume of the bone crest [15]. Tretto et al. (2019) [19] carried out a systematic review of the literature on the techniques used to prepare the bone to receive an implant and stated that osseodensification has shown promising and encouraging biomechanical results. In recent years several publications have assessed this procedure [20–24] but the occurrence and extent of peri-implant defects in the mandible have rarely been addressed. Therefore, the aim of this in vitro pilot study was to evaluate the impact of osseodensification on the increase in ridge thickness and the prevention of buccal peri-implant defects. The hypothesis was that osseodensification would expand the ridge and compress the cancellous bone around the implant site without causing major bone defects.

## Methods

### Animals

This study was approved by the Animal Ethics Committee of the Araraquara Dental School FOAr—Unesp under protocol 20/2019. Ten animals that were approximately one year old with an average weight of 100 kg and edentulous space on the jaw greater than or equal to 10 mm between the lower canine and first lower premolars were selected. The Suimartin slaughterhouse (Viana, Espírito Santo, Brazil) sacrificed the pigs to market the pork meat and provided the jaws to be used in this study. After the study the slaughterhouse disposed the mandibles in accordance with health regulations.

### Study design

After transporting the jaws to the study site, a crestal incision was performed bilaterally in the edentulous region between the lower canine and the lower first premolar. At both proximal extremities vertical buccal incisions were created to facilitate access to and visualization of the area of ​​interest. A full thickness flap exposed the bone tissue and was extended 15 mm vertically. The mandible was fixed in a device to standardize its position and bone scanning was performed with an intraoral scanner (Trios3 mono—3Shape, Copenhagen, Denmark) at the resolution determined by the manufacturer. A gauge ruler was fitted on the ridge and a digital caliper (Mitutoyo, Suzano, Brazil) was used to measure the thickness of the ridge 1 mm below the crest.

The ridge received two types of site preparation: 1—conventional with standard cutting burs (CTL group) or 2—modified by osseodensification with the Densah® burs (Densah Burs, Versah, Jackson, USA) (OD group). This was a split-mouth study, thus software (Randomizer for clinical trials, Regis Bournique) was used for randomization to determine the first side to be operated and to choose which area to receive the perforation with the cutting drills or osseodensification.

An electric motor and hand-piece (NSK, Tokyo, Japan) were used to prepare the sites to receive cone morse implants (4.5 × 10 mm CM SW Plus; S.I.N. Implant System, Sao Paulo, Brazil). In the CTL group an initial guide drill, a twist drill 2.0 and tapered burs measuring 3.0, 3.3 and 4.0 mm (S.I.N. Implant System, Sao Paulo, Brazil) were used to prepare the site perpendicularly to the ridge with the following settings: clockwise rotation at 800 RPM and 20 Ncm torque. In the OD group the pilot and osseodensification burs 2.0, 2.3, 3.0, 3.3 and 4.3 Densah® burs were used with counterclockwise rotation at 800 RPM and 20 Ncm of torque. Copious irrigation was performed to prevent bone heating while assisting in the process of cutting or densifying. The perforation depth was established at 11 mm since the implant had to be installed 1 mm below the crestal level as recommended by the manufacturer’s guidelines. The mandible was reattached to the positioning device and the digital scan was repeated to evaluate the alteration in the ridge thickness.

The implants were placed in position initially with a hand-piece operating at 20 RPM until it reached 35 Ncm torque or the crestal level. A manual torque wrench (S.I.N. Implant System, Sao Paulo, Brazil) was used to finish implant placement and to evaluate the final insertion torque. The mandibles were scanned again and the sites were verified to check the occurrence of defects in the bone walls immediately after placement. All surgical procedures were carried out by a single operator.

### Analysis of bone volume

The STL files obtained from the digital scan before (T1) and after (T2) perforation, and after implant installation (T3), were imported into software (ExoCAD, Fraunhofer-Gesellschaft, Munich, Germany) that allowed the analysis of the images and any overlap [25]. To standardize the position of every pair of evaluations, an automatic adjustment was made by the software followed by a manual fine adjustment if necessary, where the first lower premolars were used as the reference. An evaluator, blinded to groups and provided with access only to STL files, performed these measurements twice, with a 7-day interval between the first and second evaluation. The following measurements were performed: (1) T1 (baseline) linear bucco-lingual evaluation of the ridge thickness at the level (0 mm) of the bone crest and 1, 2, 4, 6 mm apically; (2) linear buccal ridge increase after T2 (T1/T2); (3) linear buccal ridge increase after T3 (T2/T3).

### Analysis of the presence and extent of bone defect

Initially, the absence of a buccal bone defect was verified after implant installation and a standardized photograph was taken. The camera was positioned on a tripod with an angulation perpendicular to the area of ​​interest and a periodontal probe (PCP-UNC 15, Hu-Friedy, Chicago, USA). In addition, the position, lens magnification and framing of each photograph was standardized using the following equipment: EOS Rebel T5i reflex camera, macro lens (EF 100 mm f / 2.8L IS USM) and circular flash Mr 14ex (Canon inc, Tokyo, Japan); the values ​​of 1/125 for shutter speed, F25 for aperture and ISO 100 were selected.

**Fig. 1:**
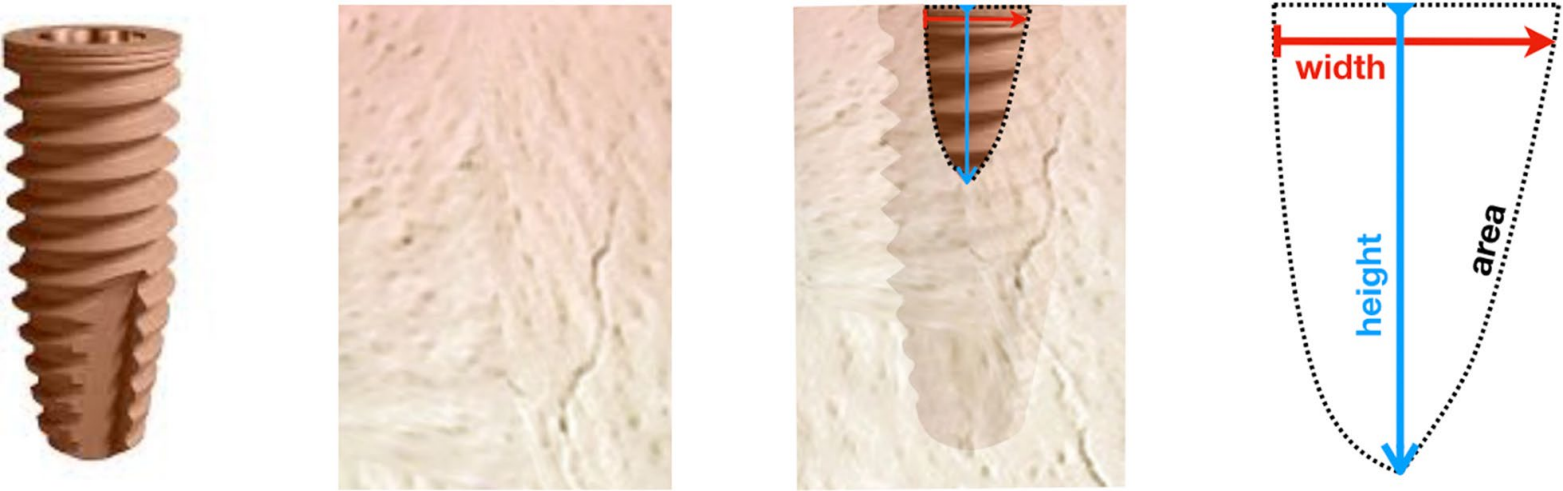
Implant design used in this study and measurement of the defect height, width and area after placement in the bone ridge

The number of buccal defects present in each group was counted. Further, the height, width, and area of ​​the defect (Fig. 1) in the buccal and lingual aspect were measured with software (ImageJ—National Institutes of Health, Bethesda, USA). Measurements in pixels were converted to approximate values in millimeters using the periodontal probe as a reference.

### Data evaluation

The height of the buccal bone defect was considered as the primary outcome of this study. Changes in defect width, area, implant insertion torque, and linear buccal ridge after implant site preparation and installation were also assessed. Non-parametric evaluations were carried out with the Mann–Whitney test to verify intergroup differences. GraphPad Prism 8 software (San Diego, CA, USA) was used to perform statistical analyses. All tests were applied with a significance level of 5% (*p* ≤ 0.05). An independent observer was aware of the group allocation at the different stages of the experiment.

## Results

Independent of the study group, the operator was able to install all implants as planned, in the desired regions. All sites were included in the study. The implant insertion torque was significantly higher in the OD group (49.9 ± 11.45 N/cm^2^) compared to the CTL group (40.4 ± 8.07 N/cm^2^), *p* < 0.05.

**Fig. 2:**
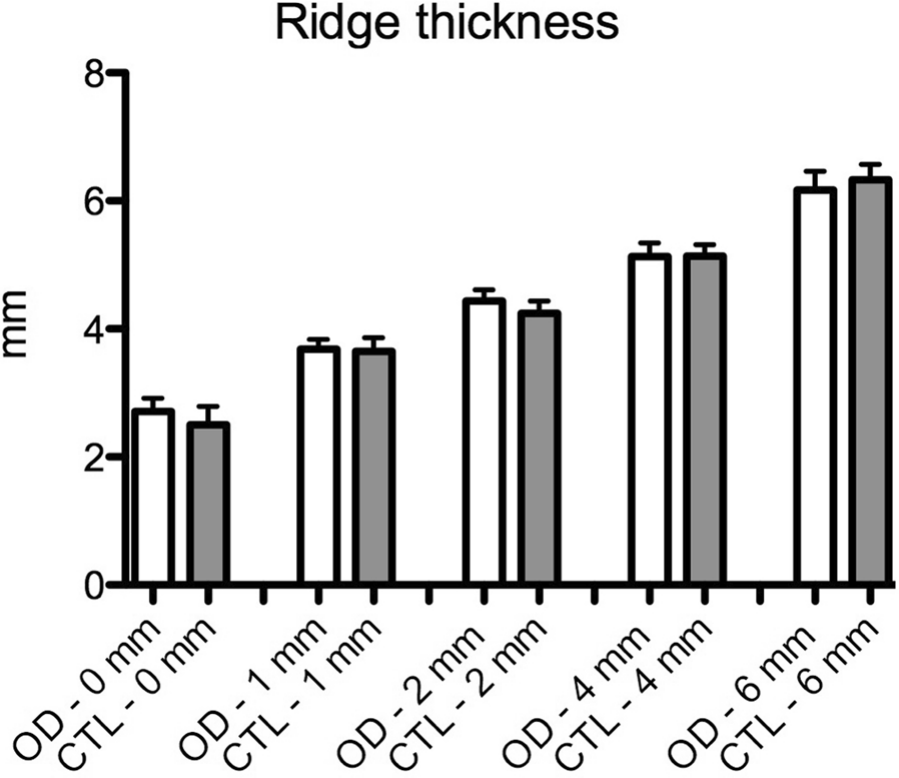
Graphic of the digital measurements, according to the group, at baseline, of the ridge thickness at the bone crest (0 mm), 1, 2, 4 and 6 mm apically

There was no significant difference between groups regarding ridge thickness in the evaluated positions, at baseline (Fig. 2). Also, there was no significant difference between the digital and analog analysis of the ridge thickness at this baseline time point.

**Fig. 3:**
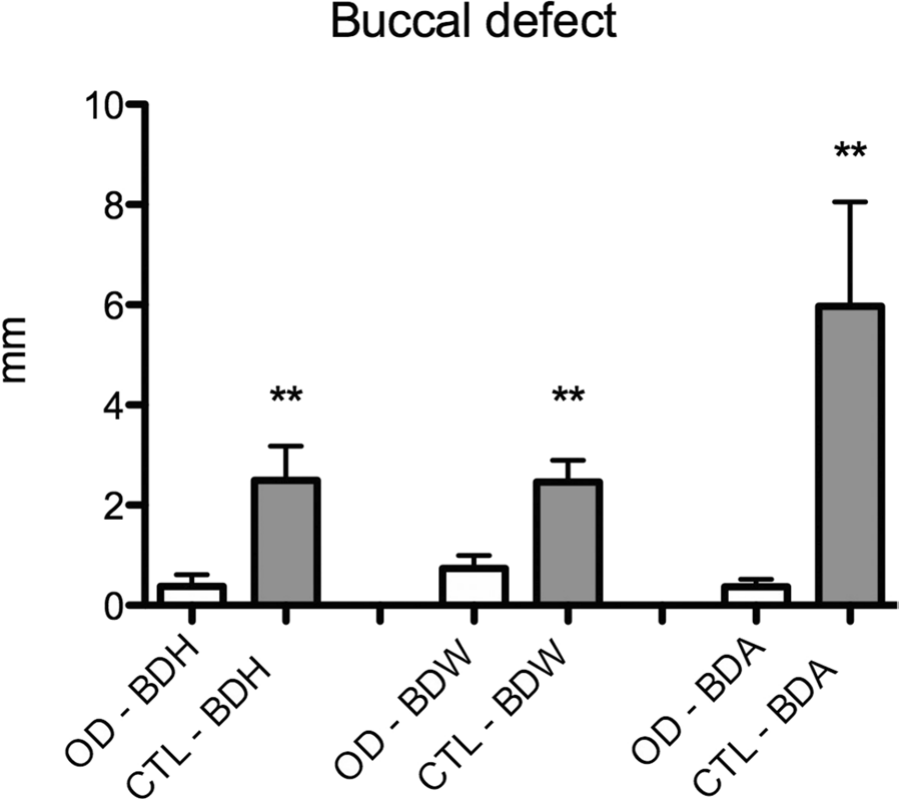
Graphic of the analysis of the buccal defect in height (BDH), width (BDW), and area (BDA) after implant placement. (*p* ≤ 0.01 in comparison to OD group. Mann–Whitney test)

**Fig. 4:**
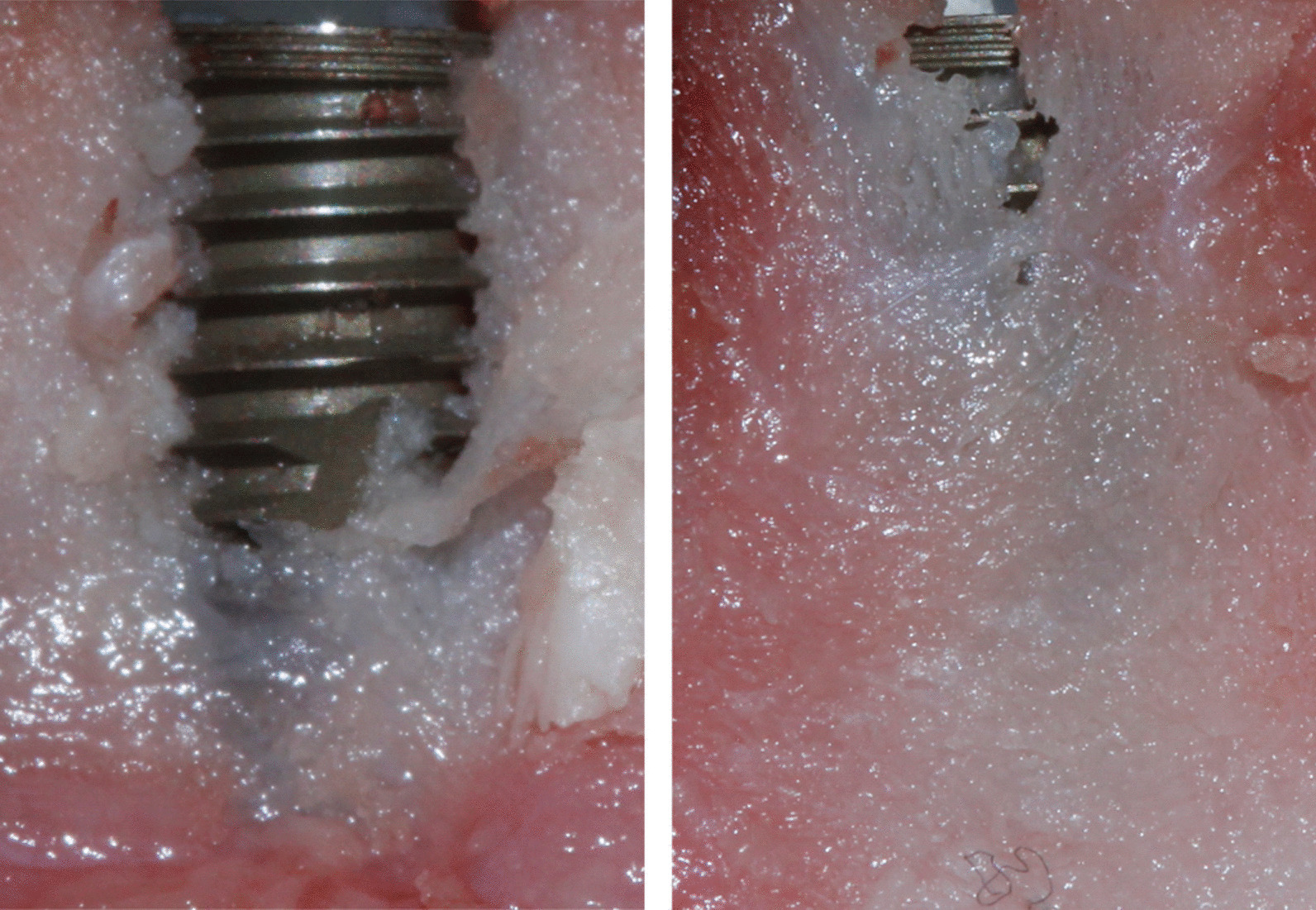
Clinical images of the worst defects in the buccal aspect of groups CTL (left) and OD (right)

**Fig. 5:**
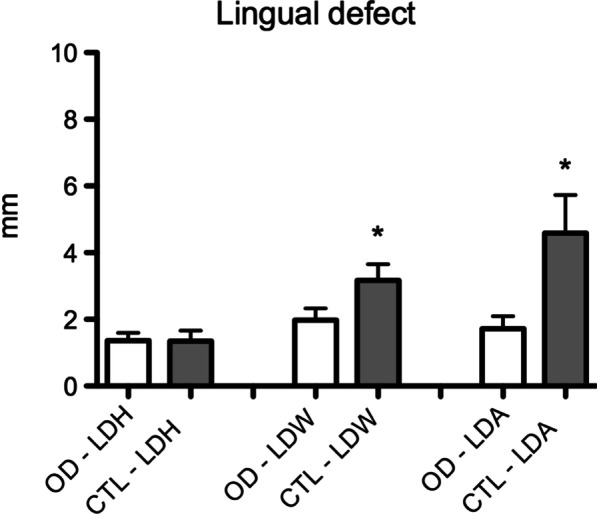
Graphic of the analysis of the lingual defect in height (BDH), width (BDW) and area (BDA) after implant placement (*p* ≤ 0.05 in comparison to OD group. Mann–Whitney test)

Osseodensification reduced the occurrence of buccal defects (Fig. 3). In the CTL group, eight buccal bone defects greater than 0.5 mm in height were present, while in the OD group, there was only one. There was a significant difference in comparison to the CTL group in the height (CTL: 2.5 ± 2.14; OD: 0.37 ± 0.72), width (CTL: 2.46 ± 1.38; OD: 0.73 ± 0.82), and area (CTL: 5.97 ± 6.57; OD: 0.36 ± 0.47) of the buccal defect (Fig. 4). In the analysis of the lingual aspect of the bone, there was a significant difference in favor of the OD group in reducing the width (CTL: 3.17 ± 1.54; OD: 1.98 ± 1.10) and area (CTL: 4.65 ± 3.24; OD: 1.72 ± 1.06) of the defect in comparison to the CTL group (Fig. 5).

**Fig. 6:**
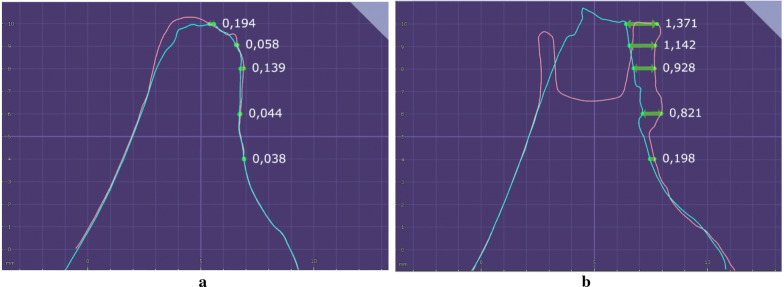
Figures exemplifying how the digital measurements were performed before (T1- blue line) and after site perforation (T2- red line) in a software to assess buccal ridge increase at the bone crest (0 mm), 1, 2, 4 and 6 mm apically. Group CTL (left) and OD (right)

**Fig. 7:**
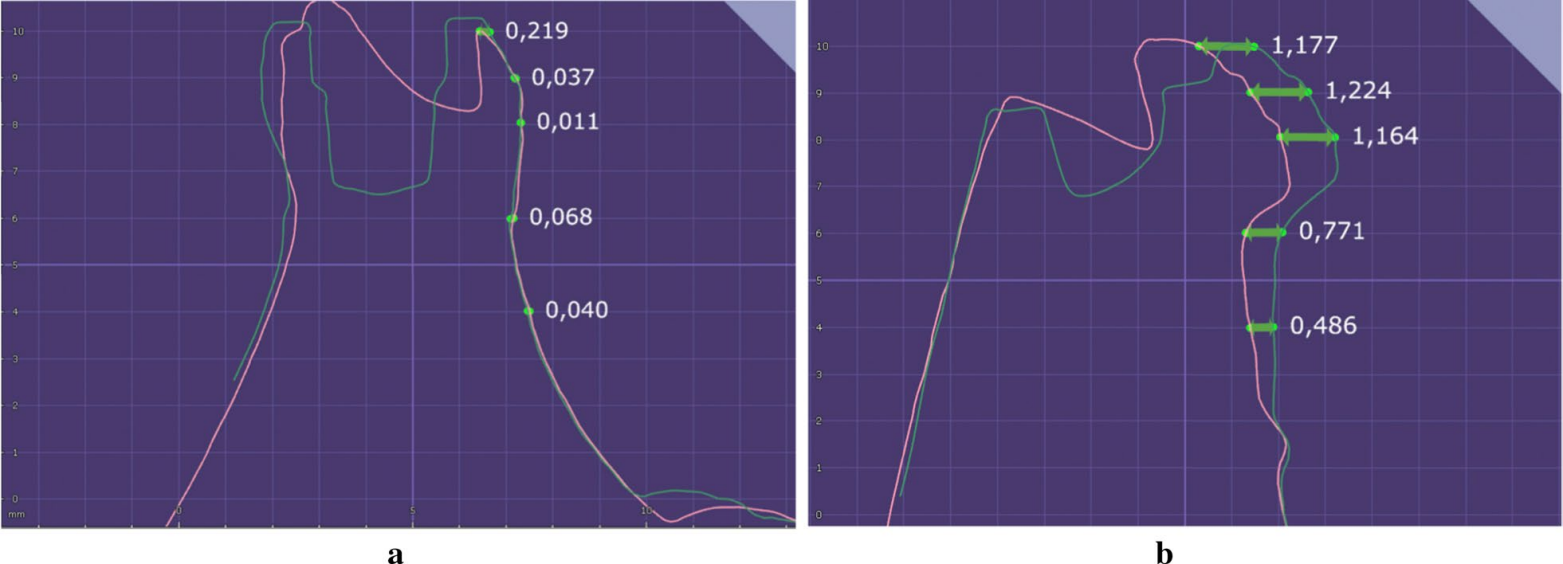
Figures exemplifying how digital measurements were performed after site perforation (T2- red line) and after implant placement (T3- green line) in a software to assess buccal ridge increase at the bone crest (0 mm), 1, 2, 4 and 6 mm apically. Group CTL (left) and OD (right)

**Fig. 8:**
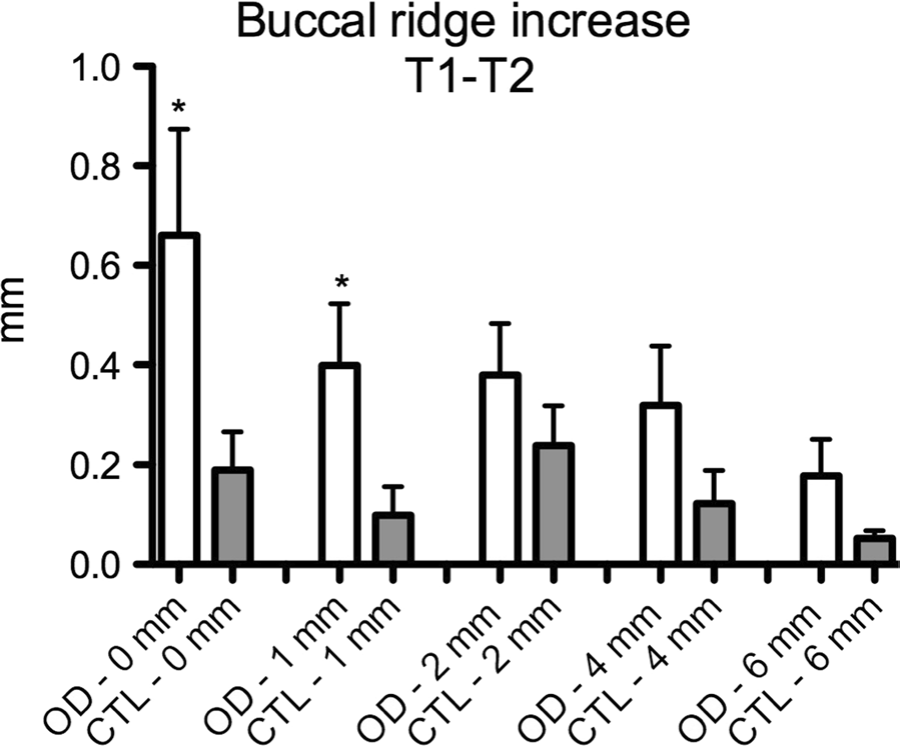
Graphic of the digital measurements compared buccal ridge increase after site perforation at the bone crest (0 mm), 1, 2, 4 and 6 mm apically (*p* ≤ 0.05 in comparison to OD group. Mann–Whitney test)

**Fig. 9:**
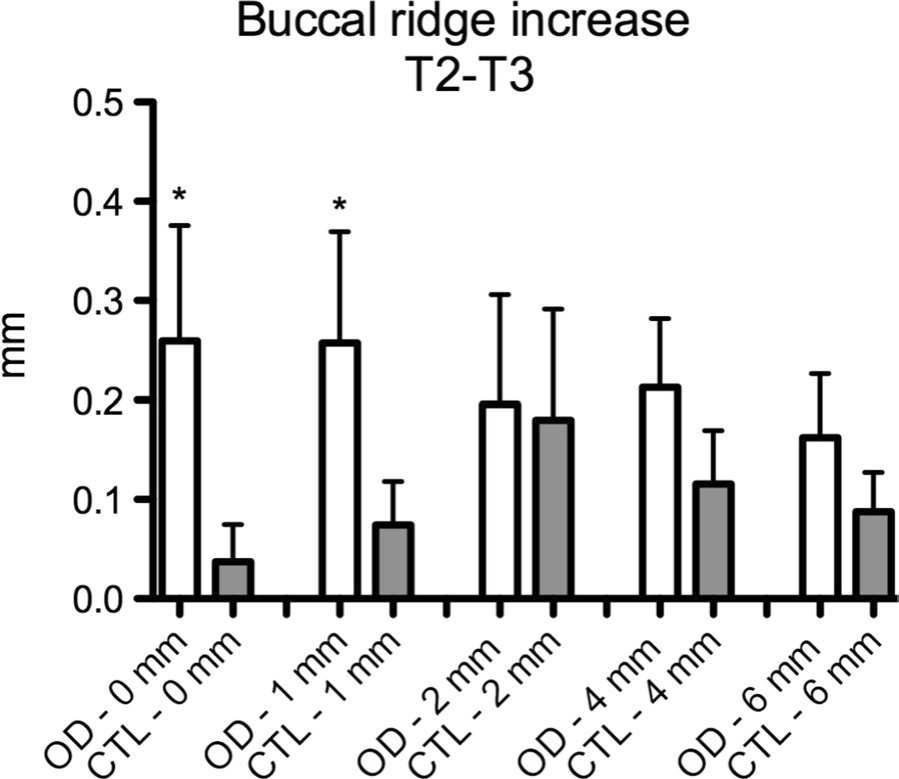
Graphic of the digital measurements compared buccal ridge increase after implant placement at the bone crest (0 mm), 1, 2, 4 and 6 mm apically* p* ≤ 0.05 in comparison to OD group. Mann–Whitney test)

The OD group presented a significant increase in ridge expansion reflected in a higher increase in the buccal bone at the crestal level and 1 mm apically after site preparation (Crestal—CTL: 0.18 ± 0.2, OD: 0.66 ± 0.64; 1 mm—CTL: 0.09 ± 0.15; OD: 0.39 ± 0.37) (T1/T2—Fig. 6) and implant placement (Crestal—CTL: 0.03 ± 0.11, OD: 0.28 ± 0.35; 1 mm—CTL: 0.07 ± 0.13; OD: 0.25 ± 0.35) (T2/T3—Fig. 7) when compared to the CTL group (Figs. 8 and 9).

## Discussion

Osseodensification led to increased ridge thickness and reduced the number and size of peri-implant buccal defects in this in vitro model. This is consistent with the literature, which describes these results as an advantage of osseodensification [15] when used in areas with lower bone density [20]. Increasing the thickness of the bone ridge can reduce morbidity and the number of surgical procedures necessary to properly install an implant in those ridges with compromised bone thickness, which impedes the proper three-dimensional positioning of the implant [1, 2].

The evolution of dental implant procedures has improved patients’ postoperative outcomes and implant survival [26, 27]. In the present study, while osseodensification was able to improve the ridge thickness, it should be acknowledged that its combination with a tapered implant also positively influenced the outcome [28]. The implant design used in this study was able to further expand the buccal bone, and also reduced the occurrence of peri-implant defects. Compared to the buccal bone wall, the expansion of the lingual bone wall was limited, as its cortical plate presented lower bone plasticity and greater thickness. Araújo et al. (2005) [29] showed that the lingual bone wall is thicker than the buccal bone wall and less prone to resorption or the formation of defects. Osseodensification promotes significant ridge expansion outcomes at sites with bone density that is below adequate [20]. In this sense, the concept of adequate has been defined with the recommendation that both cortical plates present a 1:1 ratio with a trabecular-bone core of at least 2 mm [10–15].

It has been shown that tapered implants with non-cutting threads combined with hand osteotomes are able to provide higher insertion torque and ridge expansion, especially in esthetically demanding areas [30, 31]. There are some biological, clinical and patient concerns about using such osteotomes. Ridge expansion with osteotomes may require the use of mallets, which is a more invasive and traumatic procedure and presents reduced expansive control, greater risk of bone fracture, and more patient complaints [13]. Some clinical studies have even shown that expansion with osteotomes leads to delayed healing of the implant site [32, 33]. Therefore, osseodensification has been reported to improve implant procedures in ridges with limited bone quantity or quality [19, 20].

A thin buccal bone plate has been associated with a greater risk of resorption and soft tissue recession [34]. Bone grafting may not be avoided in such situations, but osseodensification can reduce the occurrence of buccal peri-implant defects, thus allowing simultaneous implant installation and bone grafting. When the implant is surrounded by native bone, it is speculated that the healing time of the implant is shortened. A longer time would usually be needed if there was a bone dehiscence that required bone grafting involving the implant surface. Complete buccal bone regeneration after bone grafting is influenced by the site anatomy and the size of the peri-implant bone defect [8]. Osseodensification can even be performed in extraction sockets, allowing immediate implant installation with increased primary stability [35] and reducing the need for further invasive procedures. Onlay grafts performed at posterior sites to increase ridge thickness prior to implant installation have been associated with greater trauma and postoperative complications [7].

In the present study, we scanned the ridge and performed linear 3D measurements. This would be more complicated to perform in human clinical studies. Intra-oral scanning at sites that present fluids can be challenging, especially whenever there is constant bleeding [36]. However, the literature presents the results of other analyses that also support the present results. Koutozis et al. (2019) [20] performed clinical analog measurements of the alveolar ridge thickness with bone calipers pre and post osseodensification in humans and showed that it increased whenever osseodensification was performed. As shown in our study, analog evaluation can provide viable data as there were no major differences in the analog or digital evaluation of the ridge thickness 1 mm below crestal level.

Buccal and lingual bone fenestration defects are commonly found in human sockets. Nimigean et al. [37] reported that bone dehiscences were detected in almost 55% of human skulls, and the defects in the mandible accounted for more than 70% of these dehiscences. After tooth extraction, it can be expected that sockets that present loss of one of the bone walls will present reduced ridge width after healing [38]. Bone expansion provided by osseodensification is a simplified technique to improve the implant site. Osseodensification promotes alveolar ridge expansion at the osteotomy site [13], preventing bone dehiscence after implant installation.

One could speculate that cone beam computed tomography could be performed to analyze the increase in ridge volume, but there are concerns regarding the threshold needed for the detection of a thin buccal bone wall in contact with a metal implant, which often leads to significant artefacts in the images [39, 40]. This in vitro animal study presented limitations such as the lack of a sample size calculation and, since it was performed in fresh pig jaws and these could present anatomical variations in the alveolar ridge. The impact of such alterations could be reduced by a split mouth study design while providing important data about how osseodensification performs in mandibles. More clinical studies with appropriate sample size are needed to further compare the potential of osseodensification to improve clinical parameters and reduce surgical morbidity. The available data concerning osseodensification presents interesting outcomes that could lead to better clinical and biological outcomes by changing how the implant site is prepared in a simplified manner [20, 22, 24].

## Conclusion

Osseodensification increased ridge thickness by bone expansion and reduced buccal bone defects after implant installation.

## Data Availability

All data generated in this study are included in this article.

## Abbreviations

CTL: Conventional drilling with cutting burs
OD: Osseodensification with Densah® burs
